# Dysregulated Immunity to *Clostridioides difficile* in IBD Patients Without a History of Recognized Infection

**DOI:** 10.1093/ibd/izad238

**Published:** 2023-10-24

**Authors:** Laura Cook, May Q Wong, William D Rees, Alana Schick, Daniel J Lisko, Genelle R Lunken, Xiaojiao Wang, Hannah Peters, Laura Oliveira, Torey Lau, Regan Mah, Brian Bressler, Megan K Levings, Theodore S Steiner

**Affiliations:** Department of Medicine, University of British Columbia, Vancouver, BC, Canada; BC Children’s Hospital Research Institute, Vancouver, BC, Canada; Department of Microbiology and Immunology, University of Melbourne at The Peter Doherty Institute for Infection and Immunity, Melbourne, VIC, Australia; Department of Medicine, University of British Columbia, Vancouver, BC, Canada; BC Children’s Hospital Research Institute, Vancouver, BC, Canada; Department of Medicine, University of British Columbia, Vancouver, BC, Canada; BC Children’s Hospital Research Institute, Vancouver, BC, Canada; Gut4Health, BC Children’s Hospital Research Institute, Vancouver, BC, Canada; Department of Medicine, University of British Columbia, Vancouver, BC, Canada; BC Children’s Hospital Research Institute, Vancouver, BC, Canada; Department of Pediatrics, University of British Columbia, Vancouver, BC, Canada; Department of Medicine, University of British Columbia, Vancouver, BC, Canada; BC Children’s Hospital Research Institute, Vancouver, BC, Canada; Department of Medicine, University of British Columbia, Vancouver, BC, Canada; Department of Medicine, University of British Columbia, Vancouver, BC, Canada; Department of Medicine, University of British Columbia, Vancouver, BC, Canada; Department of Medicine, University of British Columbia, Vancouver, BC, Canada; Gastrointestinal Research Institute, Vancouver, BC, Canada; BC Children’s Hospital Research Institute, Vancouver, BC, Canada; Department of Surgery, University of British Columbia, Vancouver, BC, Canada; School of Biomedical Engineering, University of British Columbia, Vancouver, BC, Canada; Department of Medicine, University of British Columbia, Vancouver, BC, Canada; BC Children’s Hospital Research Institute, Vancouver, BC, Canada

**Keywords:** CD4^+^ T cell, C. difficile, microbiome, antimicrobial resistance, T cell memory

## Abstract

**Background & Aims:**

*Clostridioides difficile* is a toxin-secreting bacteria that is an urgent antimicrobial resistance threat, with approximately 25% of patients developing recurrent infections. Inflammatory bowel disease (IBD) patients are at increased risk of severe, recurrent *C. difficile* infection.

**Methods:**

To investigate a role for *C. difficile* infection in IBD pathogenesis, we collected peripheral blood and stool from 20 each of ulcerative colitis patients, Crohn’s disease patients, and healthy control subjects. We used a flow cytometric activation induced marker assay to quantify *C. difficile* toxin–specific CD4^+^ T cells and 16S ribosomal RNA sequencing to study microbiome diversity.

**Results:**

We found IBD patients had significantly increased levels of *C. difficile* toxin B–specific CD4^+^ T cells, but not immunoglobulin G or immunoglobulin A, compared with healthy control subjects. Within antigen-specific CD4^+^ T cells, T helper type 17 cells and cells expressing the gut homing receptor integrin β7 were reduced compared with healthy control subjects, similar to our previous study of non-IBD patients with recurrent *C. difficile* infection. Stool microbiome analysis revealed that gut homing, toxin-specific CD4^+^ T cells negatively associated with microbial diversity and, along with T helper type 17 cells, positively associated with bacteria enriched in healthy control subjects.

**Conclusions:**

These data suggest that IBD patients, potentially due to underlying intestinal dysbiosis, experience undiagnosed *C. difficile* infections that result in impaired toxin-specific immunity. This may contribute to the development of inflammatory T cell responses toward commensal bacteria and provide a rationale for *C. difficile* testing in IBD patients.

**Key Messages**

**What is already known?**

Inflammatory bowel disease (IBD) patients are at increased risk for recurrent *Clostridioides difficile* infection.

**What is new here?**

This is the first assessment of T cell immunity to *C. difficile* in IBD patients with no recognized history of infection, finding altered phenotypes similar to severe *C. difficile* infection in non-IBD patients.

**How can this study help patient care?**

IBD patients may have intermittent *C. difficile* infection masked by clinical overlap with IBD symptoms, and testing should be considered in patients with changes in disease activity. High levels of bacterial toxin–specific T helper type 17 cells may be a biomarker of a healthy immune response.

## Introduction

*Clostridioides difficile* infection (CDI) is the leading cause of hospital-acquired diarrhea in North America and is an urgent antimicrobial resistant threat.^1^ Most people are intermittently colonized with *C. difficile*, with approximately 40% of infants colonized in the first 12 months of life.^2^*C. difficile* growth is normally controlled by commensal bacteria; however, when commensal bacteria are altered, *C. difficile* can outgrow and produce its pathogenic toxins, TcdA and TcdB, leading to intestinal injury.^3^ Inflammatory bowel disease (IBD) patients have increased risk of CDI,^4^ and infections prevent mucosal healing and exacerbate long-term complications. The role of *C. difficile* in ongoing gut dysbiosis and inflammation in IBD patients remains undetermined and is likely underappreciated due to overlapping symptoms and the lack of tests that reliably distinguish *C. difficile* colonization from active CDI.^5^ Our previous work found that TcdB-specific CD4^+^ T cell responses distinguished CDI patients from healthy control subjects, with patients having increased antigen-specific CD4^+^ T cells yet proportionally fewer T helper type 17 (Th17) cells.^6,7^ We hypothesized that IBD patients could similarly have dysregulated immunity to *C. difficile* contributing to their dysbiosis and symptoms.

## Methods

All authors had access to the study data and have reviewed and approved the final manuscript.

### Subjects

Study protocols were approved by Clinical Research Ethics Boards of the University of British Columbia (H15-01682, H09-01238, and H18-02553) and the Vancouver Coastal Health Authority (V15-01682). All participants were ≥18 years of age and provided written informed consent with an established diagnosis of either Crohn’s disease (CD) or ulcerative colitis (UC). Exclusion criteria was immune compromise. CDI screening is routinely done for IBD patients in our clinic who present with diarrhea (symptoms suggesting an IBD flare) but not when they are asymptomatic. We did not perform any additional CDI screening in this cohort. Age- and sex-matched healthy control subjects had no history of CDI. Cohort clinical data have been previously published,^8^ and critical demographic data and additional data relevant to this study are in **Table 1**. Blood samples were collected at a single time point; 26 of 40 patients provided a stool sample at or within 1 month of blood draw and 25 of 26 tested negative for *tcdB* by polymerase chain reaction (PCR), and 1 patient who tested negative at time of sample collection had a recorded positive *tcdB* PCR test 22 months prior to enrollment. A total of 19 of 20 healthy control subjects provided stool samples and all tested negative.

**Table 1.** Characteristics of IBD patients and healthy control subjects.

ID	Age (y)	Sex	Years since diagnosis	Type of IBD treatment	Other medications	Disease score
**UC cohort**						**SCCAI**
FL001^a^	37	F	4	Methotrexate, 5-ASA	DMARD	8
FL002	62	F	22	Azathioprine, infliximab	NSAID	2
FL007	30	M	2	Azathioprine, golimumab	None	0
FL010^a^	74	F	10	5-ASA	ACE inhibitor, budesonide	1
FL011^a^	26	F	11	Azathioprine, vedolizumab	Liothyronine	8
FL012	44	F	1	Adalimumab	None	3
FL013^a^	64	M	11	5-ASA	None	1
FL014^a^	48	F	25	Adalimumab	Levothyroxine	0
FL016^a^	78	F	3	Vedolizumab	None	0
FL021	35	M	7	Infliximab	None	0
FL024^a^	43	F	15	Vedolizumab	None	5
FL027	38	M	<1	Prednisone, infliximab	Finasteride	2
FL033^a^	36	F	4	Infliximab	NDRI	0
FL034^a^	44	F	15	Azathioprine, vedolizumab, 5-ASA	None	3
FL035	27	F	4	Vedolizumab	None	1
FL036	35	M	13	Infliximab	None	1
FL037^a^	35	F	<1	Infliximab	None	2
FL038^a^	28	M	10	Infliximab	None	0
FL039^a^	25	F	4	Vedolizumab	None	2
FL040^a^	34	F	7	Infliximab	None	1
**CD cohort**						**Harvey-Bradshaw index**
FL003^a^	33	M	3	Ustekinumab	None	0
FL004^a^	70	F	30	None	ACE inhibitor, Amlodipine, NSAID	7
FL005	27	M	7	Adalimumab	None	1
FL006^a^	35	F	2	Infliximab	None	14
FL008^a^	46	M	16	Azathioprine, infliximab	None	2
FL009^a^	48	M	28	Ustekinumab	Liothyronine, Zopiclone, NDRI, TCA	2
FL015	40	M	16	None	None	2
FL017	29	M	13	Infliximab	None	2
FL018^a^	51	M	7	Prednisone, vedolizumab	None	2
FL019	48	M	7	Vedolizumab	Percocet, Quetiapine, PPI	8
FL020^a^	28	M	12	Infliximab	None	3
FL022	40	M	19	Infliximab	None	4
FL023^a^	44	M	27	Vedolizumab	SSRI	19
FL025^a^	28	F	8	Vedolizumab	None	9
FL026	39	F	18	None	Levothyroxine	15
FL028^a^	32	F	11	Hydrocortisone, infliximab	None	3
FL029^a^	37	M	10	Infliximab	Nifedipine, hydrochlorothiazide	1
FL030^a^	39	M	11	Infliximab	None	8
FL031	29	M	12	Infliximab	None	6
FL032^a^	49	M	36	Infliximab	ACE inhibitor, metformin, hydrochlorothiazide	4

For the Healthy Control (HC) cohort (n = 20), the median age was 38 (range, 24-80) years; 8 (40%) were female and 12 (60%) were male; and 13 (n = 5 female) had stool samples sequenced (median age 36 [range, 24-79] years).

Abbreviations: 5-ASA, 5-aminosalicylates; ACE, angiotensin-converting enzyme; CD, Crohn’s disease; DMARD, disease-modifying antirheumatic drugs; F, female; HC, healthy control; M, male; NDRI, norepinephrine–dopamine reuptake inhibitors; NSAID, nonsteroidal anti-inflammatory drugs; PPI, proton pump inhibitors; SCCAI, Simple Clinical Colitis Activity Index; SSRI, selective serotonin reuptake inhibitor; TCA, tricyclic antidepressants; UC, ulcerative colitis.

**Figure 1.**
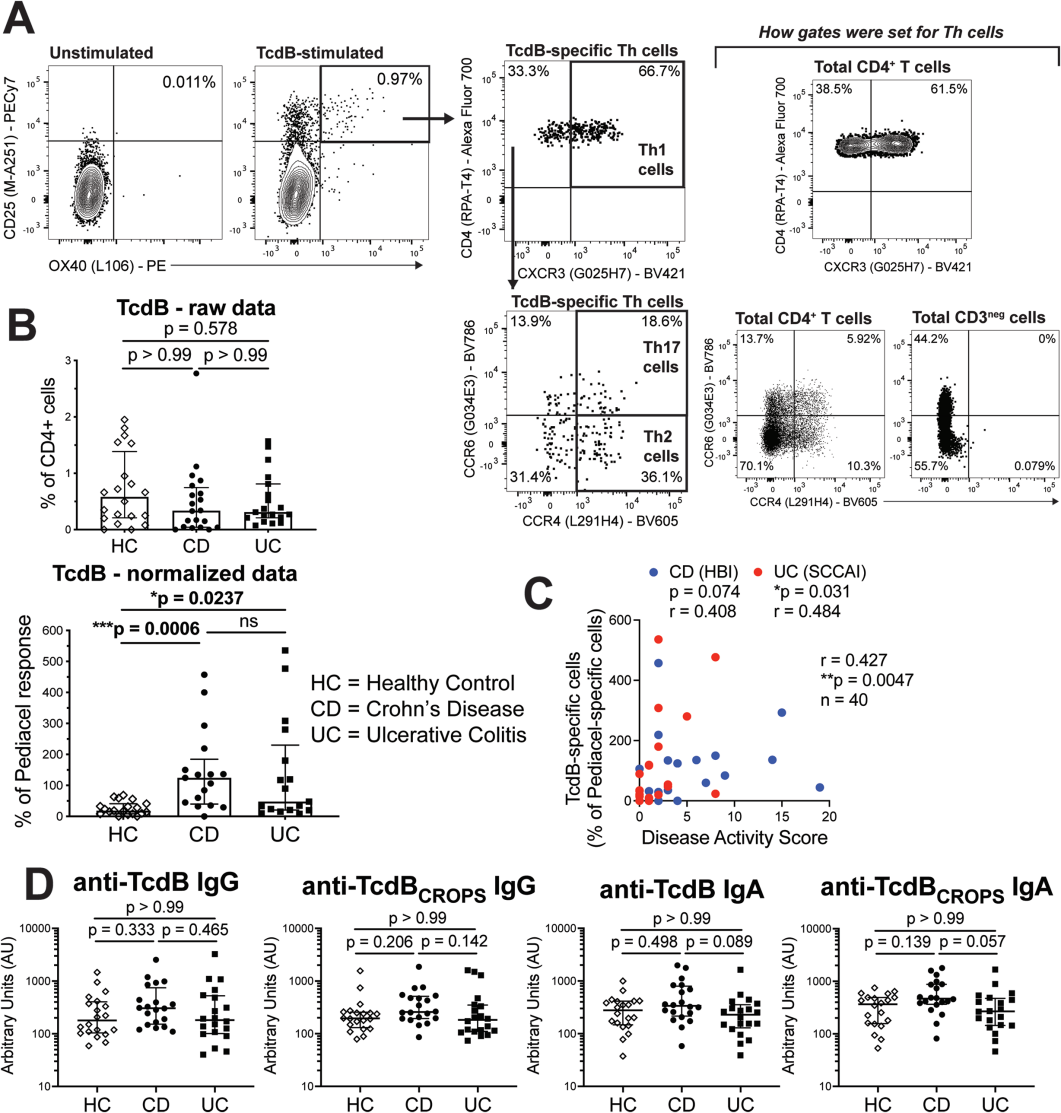
Inflammatory bowel disease patients have increased frequency of circulating TcdB-specific CD4^+^ T cells. A, Representative unstimulated and TcdB-stimulated CD25/OX40 assay wells for a Crohn’s disease (CD) patient, showing gating strategy as previously described^8^ for CD25^+^OX40^+^ cells and T helper type 1 (Th1), Th2, and Th17 cells, gates set on CD4^+^ T cells and CD3^neg^ cells. B, Comparison of TcdB-specific CD4^+^ T cell responses normalized as a percent of Pediacel vaccine responses for healthy control (HC) subjects (n = 20), CD (n = 20), and ulcerative colitis (UC) patients (n = 20). C, Spearman’s rho correlation between Harvey-Bradshaw index (HBI) for CD and Simple Clinical Colitis Activity Index (SCCAI) for UC; and TcdB-specific CD4^+^ T cells (n = 20 CD and n = 20 UC). D, Levels of anti-TcdB and anti-TcdB_CROPS_ immunoglobulin G (IgG) and IgA for cohorts in panel B measured by enzyme-linked immunosorbent assay. Statistical analysis in panels B and D used Kruskal-Wallis tests.

^a^A total of 13 of 20 CD, 13 of 20 UC patients, and 19 of 20 HC subjects provided a stool sample at time of blood draw. A total of 25 of 26 patients and 19 of 19 HC subjects were negative for *tcdB* by polymerase chain reaction. The positive result was an UC patient (FL-011) with 23.4% TcdB-specific CD4^+^ T cells (as percent of Pediacel-specific cells) with T helper type cells comprising 35.5% (**Figure 1**). One UC patient (FL-034) had a positive *tcdB* polymerase chain reaction test 22 months prior to enrolment; they had 54.17% TcdB-specific CD4^+^ T cells (as percent of Pediacel-specific cells) but with too few events to quantify Th17 cells.

### Sample Collection and Processing

Peripheral blood was collected into 9-mL sodium heparin vacutainers (BD; Biosciences) and transported at ambient temperature. CD25/OX40 assays were set up as described subsequently using whole blood within 4 hours of collection, and plasma aliquots were stored at −80 °C. Stool samples were collected into stool nucleic acid collection and preservation tubes (Norgen Biotek Corp), shipped at room temperature, and stored at −80 °C.

### Reagents

Staphylococcal enterotoxin B (SEB; Sigma-Aldrich) was used at 1 μg/mL. Pediacel, a pentavalent vaccine containing components of pertussis vaccine, diphtheria, and tetanus toxoids, inactivated poliomyelitis vaccine and *Haemophilus influenzae* B conjugate vaccine (Sanofi Pasteur Ltd) was used at 1/40 dilution. *C. difficile* TcdB toxoid (formaldehyde inactivated; List Biological Laboratories Inc) was used at 5 μg/mL.

### *C. difficile* Growth and TcdB_CROPS_ Protein Production

*C. difficile* ATCC strain 9689 was grown on YT agar plates anaerobically for 48 to 72 hours at 37 °C, then colonies were grown in BHI broth at 37 °C in an anaerobic chamber for 24 to 48 hours. Genomic DNA was extracted as previously described^9^ and TcdB_CROPS_ amplified using the following primers:

5ʹ-GGTTCGAACTATTCACTAATCACTAATTGAGC-3ʹ and
5ʹ-CTCGGATCCTGAAGAAAATAAGGTGTCACAAG-3ʹ.

TcdB_CROPS_ was expressed with a 6xHis tag in *Escherichia coli* BL21(DE3)PLysS and purified by metal affinity chromatography as previously described.^10^

### Identification of Antigen-Specific T Cells Using the CD25/OX40 Assay

The CD25/OX40 assay was performed as previously described,^8^ and the monoclonal antibody staining panel is listed in **Table 2**. A 4-laser LSRFortessa X20 flow cytometer (BD) was used for data acquisition, utilizing application settings. Assay cutoffs were >0.02% of CD4^+^ T cells (which is the mean + 3 SD of unstimulated wells) and consisting of at least 20 cells. Analysis was performed using FlowJo software (version 10.6.1; BD) with DownSample v3.0.0, tSNE v2.0,^11^ and FlowSOM v1.5 plugins.^12^

**Table 2.** Monoclonal antibody panel used for CD25/OX40 assay analysis.

Specificity	Conjugation	Clone	Amount per 200-μL assay well (µL)	Supplier	Cat. number
CD3	Brilliant Violet 510	SK7	0.5	BioLegend	344828
CD4	Alexa Fluor 700	RPA-T4	1	BD	557922
CD25	PECy7	M-A251	5	BD	557741
CD134(OX40)	PE	L106	20	BD	340420
CD39	FITC	A1	2	BioLegend	328206
PD-1	APCeFluor780	eBioJ105	2	eBioscience	47-2799
Integrin β7	PECy5	FIB504	2	BD	551059
TIGIT	Alexa Fluor 647	A15153G	1	BioLegend	372724
CXCR3	Brilliant Violet 421	G025H7	2	BioLegend	353716
CCR4	Brilliant Violet 605	L291H4	1	BioLegend	359418
CD226	Brilliant Violet 711	DX11	2	BD	564796
CCR6	Brilliant Violet 785	G034E3	2	BioLegend	353422

### Enzyme-Linked Immunosorbent Assay

Enzyme-linked immunosorbent assay (ELISA) was used to quantify anti-TcdA, TcdB, immunoglobulin G (IgG), and IgA levels in plasma as previously described,^6^ using plates coated with 1 µg/mL of TcdA, TcdB, or TcdB_CROPS_ or 1/100 dilution of Pediacel. A standard of pooled high-titer plasma generated relative arbitrary units for cross-assay standardization.

### Quantitative PCR Detection of *C. difficile* TcdB

DNA was isolated from stool samples as previously described^8^ and 100 ng DNA used for PCR analysis. Primers and probe to amplify *C. difficile tcdB* were (all listed as 5ʹ to 3ʹ): Forward primer: GAAAGTCCAAGTTTACGCTCAAT; Reverse primer: GCTGCACCTAAACTTACACCA and Probe 6-FAM/ACAGATGCA/ZEN/GCCAAAGTTGTTGAATT/IABkFQ. An internal control reaction amplified a linearized recombinant plasmid containing murine genomic sequences (p5Pluc) using: Forward primer: TTGGTTCCAGGCATGAGTTTG; Reverse primer: CATGAAAGCTGCACAACTCCC and Probe: *HEX*/CTGGATGAC/*ZEN*/TTTGAGGTCAAGGCA/*IABkFQ* (Integrated DNA Technologies). The *tcdB* positive control was DNA extracted from a TcdB^+^*C. difficile* strain provided by the clinical diagnosis lab at Vancouver General Hospital, Vancouver, Canada. Quantitative PCR was performed on Applied Biosystems 7500 Fast Real-time PCR system using PrimeTime Gene Expression Master Mix (Integrated DNA Technologies) and cycling conditions of 3 minutes at 95 °C followed by 50 cycles of 95 °C for 10 seconds, 60 °C for 30 seconds.

### 16S Ribosomal RNA Sequencing

DNA was extracted from stool samples, quantified and sequenced as described previously.^1^ FASTQ files were processed using a custom script based on the R package Dada2 version 1.20.0.^13^ Reads were truncated at 200 bp (forward) and 80 bp (reverse) and quality-filtered using an expected error (EE = sum(10^(-Q/10)) threshold of 2 and 3 for forward and reverse reads, respectively. Following read merging and chimeric sequence removal, a table of amplicon sequence variants was generated with taxonomic assignment using the Silva database (version 138). These data have been uploaded to National Center for Biotechnology Information BioProject (ID: PRJNA991428) and can be accessed at https://www.ncbi.nlm.nih.gov/bioproject/991428.

### Statistics

Statistical analyses between 2 groups used Mann-Whitney *U* test or, for paired samples, Wilcoxon signed rank test. Analysis of ≥3 groups used Kruskal-Wallis 1-way analysis of variance or, for paired samples, a Friedman 1-way analysis of variance with Dunn’s multiple comparison posttest. Correlation analyses calculated Spearman’s rho (r). *P* values were considered significant when <.05 (unless specified otherwise). Prism version 8 (GraphPad Software) and R (version 4.1.1; R Foundation for Statistical Computing) were used for all statistical analyses. Error bars represent median ± interquartile range (IQR); *P ≤ .05, **P ≤ .01, ***P ≤ .001, ***P ≤ .0001, and ns indicates not significant. Alpha diversity was estimated using species richness and the Shannon index of diversity on raw counts of amplicon sequence variants. Associations between immune variables and alpha diversity were determined using Spearman rank correlation analysis. Principal coordinates analysis on a Bray-Curtis distance matrix were used to determine beta diversity, with permutational multivariate analysis of variance (R package Adonis) used to test for statistical differences of sample composition. DESeq2 (version 1.32.0)^14^ was used to identify differentially abundant amplicon sequence variants based on disease state, and variants with *P* < .01 were considered significant.

## Results

To examine a role for impaired *C. difficile* immunity in IBD pathogenesis, we analyzed proportions and phenotype of toxin B(TcdB)-specific CD4^+^ T cells and levels of anti-toxin IgG and IgA antibodies. Blood was collected from CD (n = 20) and UC (n = 20) patients and age- and sex-matched healthy control subjects (n = 20). No participants were taking antibiotics, and only 1 had a clinical history of CDI (UC patient FL-034), with a positive PCR test recorded 22 months prior to enrollment in this study. Stool samples were available for 19 of 20 healthy control subjects, and all tested negative for *tcdB* by PCR. For 26 of 40 patients that provided stool samples, 25 of 26 tested negative for *tcdB* by PCR (**Table 1**), with the positive result (UC patient FL-011) having no clinical history of infection. The 2 patients with history of CDI, or positive PCR result, were not excluded from analysis, and their individual results along with cohort characteristics are listed in **Table 1**.

We performed CD25/OX40 antigen induced marker assays^6-8,15^ by incubating peripheral blood with antigen for 44 hours, and quantified antigen-specific CD4^+^ T cells by induced coexpression of CD25 and OX40 (**Figure 1A** and **Table 2**). The TcdB-specific T cells as a percent of total CD4^+^ T cells were not different between cohorts (**Figure 1B**). However, we previously reported for this IBD cohort that CD4^+^ T cell responses to both a mitogen stimulus (Staphylococcal enterotoxin B) and the pentavalent childhood vaccine Pediacel were significantly reduced compared with healthy control subjects.^8^ Our IBD cohort had an inverse correlation between the proportions of Pediacel-specific CD4^+^ T cells, but not Staphylococcal enterotoxin B–stimulated CD4^+^ T cells, and age.^8^ As healthy control subjects were matched by age (and sex), the reduced frequency of vaccine antigen-specific CD4^+^ T cells in IBD patients was not attributed to age-related changes in immune functions. This indicates the reduced CD4^+^ T cell memory responses in these IBD cohorts is most likely due to treatment/disease-induced immune suppression, meaning that the frequency of TcdB-specific CD4^+^ T cells requires normalization. Therefore, we propose Pediacel vaccine responses are the best means to normalize for treatment/disease-induced changes in a pre-existing memory cell population that accounts for age-related decline.

Upon normalization to the Pediacel response, we found that TcdB-specific CD4^+^ T cells were significantly increased in CD (*P* = .0006) and UC patients (*P* = .0237) compared with healthy control subjects (**Figure 1B**). We also detected a weak positive correlation between disease activity scores, being the Harvey-Bradshaw index for CD and Simple Clinical Colitis Activity Score for UC, and normalized proportions of TcdB-specific CD4^+^ T cells, indicating that milder symptoms were associated with a reduced proportion of memory TcdB-specific CD4^+^ T cells in peripheral blood (*r* = 0.427, *P*** = **.0047) (**Figure 1C**). Notably, there was no difference in the levels of anti-TcdB or anti-TcdB_CROPS_ (the C-terminal combined repetitive oligopeptides domain of TcdB, a target of neutralizing antibodies)^16^ IgG or IgA between IBD patients and control subjects (**Figure 1D**), further confirming that TcdB-specific CD4^+^ T cells are more relevant than antibodies in defining normal vs pathologic immunity to TcdB.^6^ However, similar to our findings for anti-flagellin antibodies in these cohorts,^8^ anti-TcdB IgA levels were decreased in UC compared with CD patients. Future studies should investigate the presence of TcdB-specific CD8^+^ T cells and levels of TcdB-stimulated IgA and IgG release to assess whether there are dysfunctional relationships, or whether only CD4^+^ T cells are responding to potentially low levels of toxins secreted during episodes of undetected *C. difficile* outgrowth.

**Figure 2.**
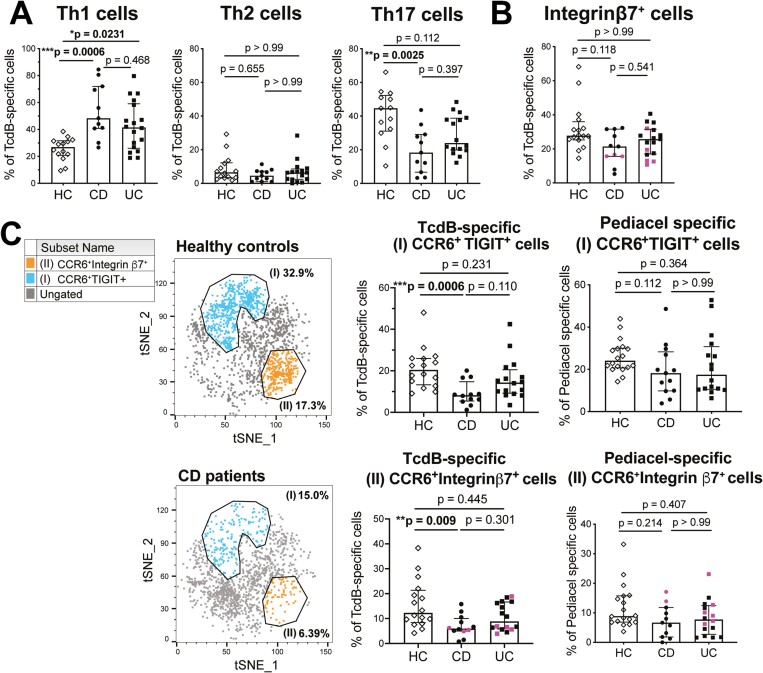
Inflammatory bowel disease patients have a reduced frequency of TcdB-specific T helper type 17 (Th17) cells. A, Analysis of TcdB-specific T cell proportions of Th1 cells (n = 14 healthy control [HC] subjects, n = 11 Crohn’s disease [CD] patients, n = 18 ulcerative colitis [UC] patients), Th2 cells (n = 14 HC, n = 11 CD patients, n = 16 UC patients), and Th17 cells (n = 13 HC subjects, n = 11 CD, n = 16 UC). B, Proportions of integrin β7^+^ cells within TcdB-specific responses for n = 12 CD and n = 17 UC. C, TcdB-specific CD4^+^ T cells were analyzed using unbiased clustering analysis (FlowSOM). Two clusters with statistically significant differences between cohorts after individual hierarchical gating analyses are shown on tSNE plots. Proportions of each cluster within TcdB-specific CD4^+^ T cells are shown for all groups (n = 16 HC subjects, n = 12 CD patients, n = 16 UC patients) and compared with proportions within Pediacel-specific responses. Statistical analysis used Kruskal-Wallis tests, patients receiving vedolizumab are colored pink in all plots.

We have previously shown that TcdB-specific cells sorted from this CD25/OX40 activation assay proliferate to TcdB antigen stimulus and secrete Th17 cell associated cytokines.^6^ Our phenotyping analysis found that the proportion of TcdB-specific Th17 cells was decreased in some UC patients (*P* = .112, bimodal distribution) and significantly decreased in the CD patient cohort compared with healthy control subjects (*P*** **= 00025) (**Figure 2A**). This is despite, as previously published, CD patients having significantly higher amounts of circulating Th17 cells as a proportion of unstimulated CD4^+^ T cells than healthy control subjects.^8^ Of note, circulating Th1 cell frequencies as a proportion of unstimulated CD4^+^ T cells were not different between cohorts.^8^ There was also a significant increase in TcdB-specific Th1 cells in CD and UC patients compared with healthy control subjects (*P *= .0006 for CD and UC patients vs *P* = .0231 for healthy control subjects) (**Figure 2A**), but there were no differences for Th2 cells. Our previous studies of TcdB responses in non-IBD patients with recurrent CDI found a significant reduction in the Th17 cell proportion of the TcdB-response in patients with recurrent infection compared with new-onset infection.^6^ In this earlier study, non-IBD patients with recurrent CDI had a median Th17 cell proportion of the total TcdB-response of 32.8% (IQR, 28.8%-45.6%). In the present study, the median Th17 cell proportion in UC patients was 24% (IQR, 19.8%-38.7%) and in CD patients was 18.3% (IQR, 6.7%-29.1%). So, although these data are from different studies, the comparison indicates that chronic antigen exposure skews the Th repertoire to have proportionally fewer Th17 cells, and this is further exacerbated in the setting of IBD.

The proportion of TcdB-specific CD4^+^ T cells expressing the gut homing marker integrin β7 was not statistically different between cohorts, and this did not change if the 4 CD and 6 UC patients (shown in pink) receiving the anti-integrin β7 monoclonal antibody vedolizumab were excluded from analysis (**Figure 2B**). Altered TcdB-specific responses were further confirmed by FlowSOM clustering analysis. We found 2 cell clusters with significant differences between patients and control subjects that both expressed the Th17-associated marker CCR6 (**Figure 2C**). Cluster I coexpressed CCR6 and the co-inhibitory receptor TIGIT (T cell immunoreceptor with Ig and ITIM domains), and cluster II coexpressed CCR6 and integrin β7. Both populations were significantly decreased in CD patients compared with healthy control subjects (*P*** **= .0006 vs *P*** **= .009) (**Figure 2C**), even when patients receiving vedolizumab were excluded from analysis. There were no differences in the proportions of either CCR6^+^TIGIT^+^ cells or CCR6^+^integrin β7^+^ cells within Pediacel-specific responses, confirming that the reduction in these cells is unique to the TcdB-specific cells (**Figure 2D**). These data suggest that expression of the gut homing marker integrin β7 is important for protective cellular immunity to *C. difficile*.

**Figure 3.**
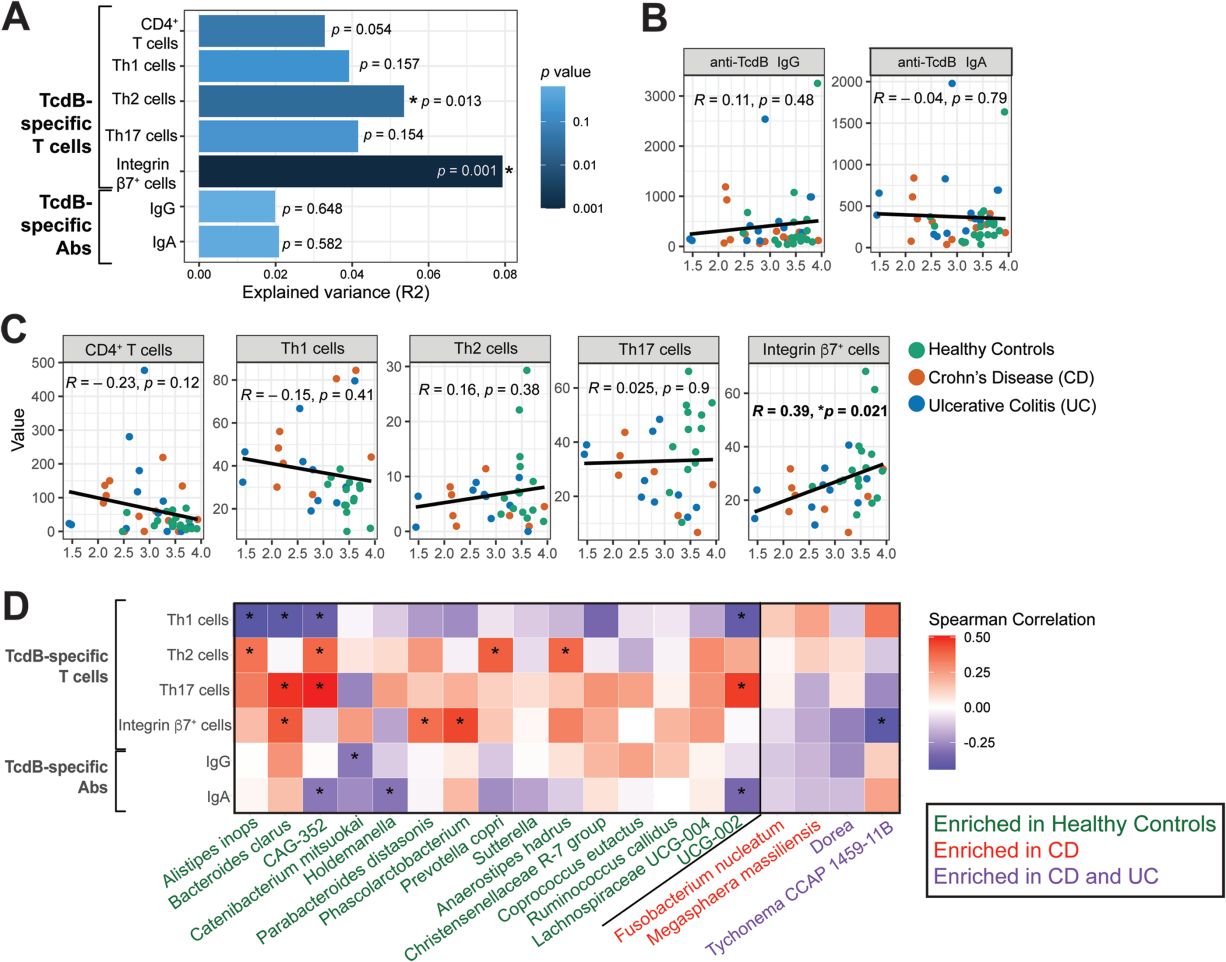
Correlations of TcdB-specific immune responses and microbiome. A, Proportion of variation in overall microbiome composition (R^2^) explained by the y-axis variable using permutational multivariate analysis of variance. Spearman rank correlations with microbiome alpha diversity (estimated using Shannon index) and TcdB-specific (B) antibodies and (C) T cells (n = 39; being n = 13 from each cohort). D, Heat map showing Spearman correlation analyses between the relative abundance of differentially expressed bacterial taxa and anti-TcdB immune responses (n = 39). *P < .05. Ab, antibody; CD, Crohn’s disease; Ig, immunoglobulin; Th, T helper; UC, ulcerative colitis.

Next, we investigated associations between proportions of TcdB-specific T cells and IgG/IgA and stool microbiome using 16S ribosomal RNA sequencing of 13 samples from each cohort. Our previous analyses of these data found that, compared with healthy control subjects, IBD patients had significantly reduced Shannon diversity, which had a significant negative correlation with their disease activity score and CD4^+^ T cell responses to bacterial flagellin.^8^ Here, we investigated associations between adaptive immune responses to TcdB and overall microbiome beta diversity (being a measure of variance between individuals), with a principal component analysis plot of these data already published.^8^ We found that the frequency of TcdB-specific CD4^+^ T cells accounted for approximately 3% variance (*P* = .054), while the TcdB-specific integrin β7^+^ (gut homing) cells accounted for 8% variance (*P* = .001), and Th2 cells accounted for 5% variance (*P* = .013) (**Figure 3A**). The TcdB-specific TIGIT^+^, PD1^+^, and CD39^+^ T cell subsets and anti-TcdB_CROPS_ antibodies did not significantly contribute to explained microbial variance and were not included in further correlation analyses (data not shown). Stool analysis included 3 of 13 CD and 5 of 13 UC patients, being a total of 8 of 26 IBD patients receiving vedolizumab. Only 3 of 8 of these patients receiving vedolizumab had TcdB-specific integrin β7^+^ cell proportions falling in the 25th percentile and 1 of 8 was in the 75th percentile of the IBD cohort, so this treatment is unlikely to explain the association of integrin β7^+^ cells with microbial variance.

We next conducted correlation analysis between immune responses and microbiome alpha diversity (being a measure of the microbial diversity within an individual). There were no correlations with toxin-specific IgG or IgA (**Figure 3B**), but TcdB-specific integrin β7^+^ cells had a significant positive correlation (*r* = 0.39, *P* = .021) (**Figure 3C**). Finally, we used pairwise DESeq2 analysis to identify differentially abundant bacterial taxa cohorts, then performed a correlation analysis between their relative abundance and TcdB-specific immune responses. In general, bacteria enriched in healthy control subjects (*Alistipes inops*, *Bacteroides clarus*, *CAG-352*, *Catenibacterium mitsuokai*, *Holdemanella, Parabacteroides distasonis*, *Phascolarctobacterium*, *Prevotella copri*, *Anaerostipes hadrus*, and *UCB-002*) had significant positive correlations with TcdB-specific Th17, Th2, and integrin β7^+^ cells and negative correlations with TcdB-specific Th1 cells and IgG/A (**Figure 3D**). TcdB-specific integrin β7^+^ cells also had a significant negative association with *Tychonema CCAP 1459-11B*, which was enriched in both CD and UC patients. Of note, this species of cyanobacteria produces a neurotoxin, anatoxin-a, that is harmful to humans.^17^ These data support a beneficial role for gut homing (integrin β7^+^) TcdB-specific CD4^+^ T cells in IBD patients. Further studies incorporating longitudinal microbiome sampling along with CDI screening and metabolomic analyses are required to investigate whether the bacterial taxa positively correlated with TcdB-specific immune responses in healthy participants play a role in colonization resistance and/or enhancing *C. difficile* specific immune responses.

## Discussion

We previously reported that IBD patients have increased flagellin-specific CD4^+^ T cells compared with healthy control subjects but reduced Th17 cell proportions within these responses,^8^ paralleling observations for TcdB-specific CD4^+^ T cells in recurrent CDI patients.^6^ Notably, this was not the case for responses to Pediacel,^6^ indicating the gut antigen-specific nature of the relative reduction in Th17 cells. We now extend these findings to TcdB-specific CD4^+^ T cells in IBD patients with no clinical history of *C. difficile* infection. These data highlight that reduced proportions of bacterial antigen-specific Th17 cells, for both commensal and pathogenic organisms, are associated with disease severity.

Although IBD patients are known to have increased risk of acquiring CDI and of severe, complicated infections,^4^ routine screening for active CDI is not currently recommended, due to the inability to confidently distinguish colonization from disease.^5^ In our cohorts, there was no clinical history of CDI, and all but 1 patient that provided a stool sample tested negative for CDI by *tcdB* PCR; however both CD and UC patients had increased circulating TcdB-specific CD4^+^ T cells. Moreover, in IBD patients, gut homing TcdB-specific cells had statistically significant associations with microbiome variance between patients (beta diversity), within patients (alpha diversity), and with bacterial species that were differentially abundant in healthy individuals.

These data are from small cohorts (n = 20) with large variability in disease activity and treatment, and our findings need to be replicated in a larger cohort, ideally excluding patients receiving vedolizumab. While the results of this cohort study cannot be used to direct clinical practice without further validation, our data support the hypothesis that TcdB-specific CD4^+^ T cell responses might be useful in identifying IBD patients who would benefit from microbiome-based therapeutics to reduce *C. difficile* vegetative growth and toxin production. Our data also provide a rationale for additional studies to evaluate the impact of *C. difficile* infection on incidence of disease in individuals at high risk of acquiring IBD (i.e., siblings). Studies should also investigate the impact of more regular *C. difficile* testing, and treatment of any infection, in newly diagnosed IBD patients.

## Conclusions

Together, our data support a beneficial role, and possibly a requirement, for Th17 cells and cells expressing the gut homing marker integrin β7 in protective immunity to *C. difficile* that may help explain the failure of drugs targeting IL17A to provide benefit in IBD.^18^ Further research should explore contributions of *C. difficile* overgrowth and low-level toxin production to dysbiosis, gastrointestinal symptoms and perturbations in the memory CD4^+^ T cell repertoire in IBD patients. It must also be noted that our findings from peripheral blood may be significantly different to the gut. Given that recruitment of T cells from blood to mucosa is almost certainly occurring in IBD patients, analysis of biopsy specimens is critical to determine function and disease relevance of TcdB-specific T cells in the gut.
